# Neurodegenerative Diseases: What Can Be Learned from Toothed Whales?

**DOI:** 10.1007/s12264-024-01310-2

**Published:** 2024-11-01

**Authors:** Simona Sacchini

**Affiliations:** Department of Morphology, Universidad de Las Palmas de Gran Canaria (ULPGC), Campus Universitario de San Cristóbal, c/ Blas Cabrera Felipe s/n, 35016 Las Palmas de Gran Canaria, Spain

**Keywords:** Neurodegenerative diseases, Neurodegeneration, Alzheimer’s disease, Amyloid β, Natural animal models, Cetaceans, Toothed whales

## Abstract

Neurodegeneration involves a wide range of neuropathological alterations affecting the integrity, physiology, and architecture of neural cells. Many studies have demonstrated neurodegeneration in different animals. In the case of Alzheimer's disease (AD), spontaneous animal models should display two neurohistopathological hallmarks: the deposition of β-amyloid and the arrangement of neurofibrillary tangles. However, no natural animal models that fulfill these conditions have been reported and most research into AD has been performed using transgenic rodents. Recent studies have also demonstrated that toothed whales - homeothermic, long-lived, top predatory marine mammals - show neuropathological signs of AD-like pathology. The neuropathological hallmarks in these cetaceans could help to better understand their endangered health as well as neurodegenerative diseases in humans. This systematic review analyzes all the literature published to date on this trending topic and the proposed causes for neurodegeneration in these iconic marine mammals are approached in the context of One Health/Planetary Health and translational medicine.

## Introduction

The suborders Mysticeti (baleen whales) and Odontoceti (toothed whales) make up the entire order Cetacea. The only mammalian groups that have perfect aquatic adaptations are the order Cetacea and the manatee family (order Sirenia). All cetaceans are carnivores, but their diet is diversified between the two suborders and among the different families. Certain species of toothed whales (TWs), like sperm or beaked whales, hunt at great depths and utilize food sources that are inaccessible to other predators. While TWs eat a diversified diet of benthic invertebrates—squid, fish, seabirds, and other marine mammals—baleen whales have the most monotypic diets and are specialized in zooplankton [1]. TWs can effectively feed in choppy sea waters, where visually dominant species cannot effectively forage because they use echolocation, an adaptation that helps them orient and forage in conditions with limited visual cues [2]. While the lactation of TWs is normally significantly longer—lasting 1–3 years—many baleen whales have relatively short lactation—5-7 months [3].

**Fig. 1:**
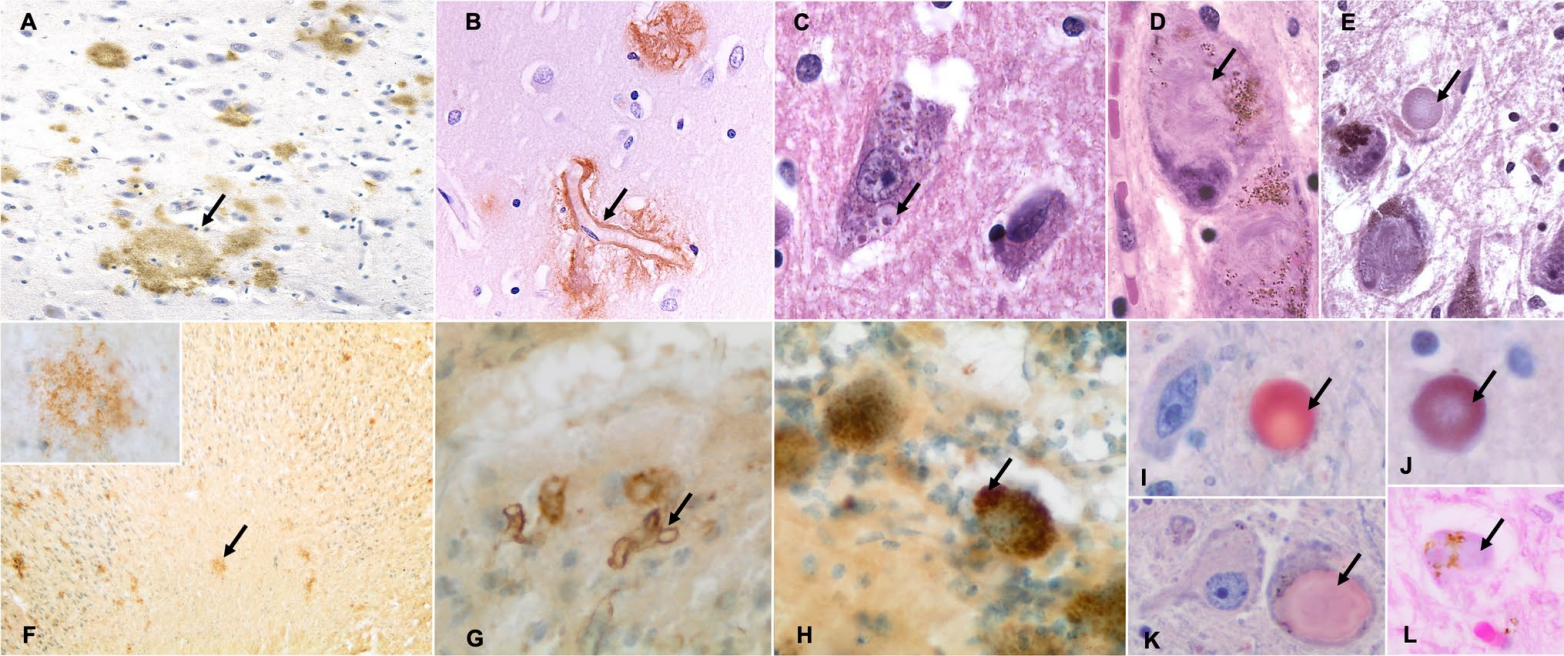
Comparative histopathological features of the most representative NDD hallmarks in humans and TWs. Images **A**–**E** are from ^[104]^; Permission to reuse the images directly has been granted by Springer. Images were taken by Rob A.I. de Vos, a neuropathologist at the Laboratorium Pathologie OostNederland (LabPON). Images **F**-**J**,**L** are from ^[18,21,86]^; manuscripts are open access and no special permission is required to reuse figures. **A** Aβ-deposits (plaques) in a person with AD. **F** Aβ-deposits (plaques) in an aged Atlantic spotted dolphin. Aβ-immunostain, Nissl counterstain. **B** Vascular Aβ deposition (capillary amyloid angiopathy) in capillaries in the human brain. **G** Vascular Aβ deposition in the amygdaloid body of an Atlantic spotted dolphin. Aβ-immunostain, Nissl counterstain. **C** GVD in the human brain. Hematoxylin–Eosin stain. **H** GVD in the Purkinje cells of a Blainville’s beaked whale. NFT-immunostain, Nissl counterstain. **D, E** Globose NFT in the human substantia nigra. Hematoxylin–Eosin stain. **I** alpha-synuclein immunopositive round body in the neuropil of the substantia nigra of a short-finned pilot whale. **J** Ubiquitin-immunopositive round body in the neuropil of the substantia nigra of the same animal. **K** A globose alpha-synuclein-immunonegative inclusion in a neuron of the substantia nigra in the same case. **L** Round pale basophilic inclusion in the perikaryon of a neuron of the locus caeruleus, pushing neuromelanin to the periphery. Blainville’s beaked whale, Hematoxylin–Eosin stain.

Recent investigations have shown that TWs exhibit neuropathological traits that may help us comprehend both the neurodegenerative diseases (NDDs) that affect humans as well as the marine mammals’ deteriorating health. Alzheimer's disease (AD) is intricate and multifaceted, as well as the most common NDD. Numerous genetic, environmental, and lifestyle risk factors have been found for AD [4]. Beta-amyloid (Aβ) deposition, as senile or neuritic plaques (SPs) and cerebral amyloid angiopathy (CAA), and hyperphosphorylated tau, in the form of neurofibrillary tangles (NFTs), are two neurohistological characteristics of AD that should be replicated in spontaneous animal models (Figure 1). Indeed, tau accumulation occurs before Aβ pathology, but once it reaches a threshold concentration, Aβ pathology appears to promote the spread of tau aggregates and neuronal death [5].

However, there are no known natural animal models that meet these requirements, so transgenic rodents have been used in the majority of studies on AD. The literature reveals evidence of features of human NDDs in different animal species but there is a significant difference from human disease as no natural model—except for some non-human primates—shows the twin hallmarks of AD (plaques and tangles), neuronal loss, and cognitive impairment. In fact, although conventional NFTs have been documented in cats and pinnipeds [6], only significant τ phosphorylation without characteristic NFTs has been recorded in sheep and goats (*Cetartiodactyla*), cats, dogs, leopards, cheetahs, bison, degu, wolverines, bears, and American bison. In these situations, τ alterations are typically random and do not take on "full-blown" traits similar to those seen in humans [7].

Transgenic mice display a mutant type I transmembrane protein called amyloid precursor protein (APP), attributable in large part to consecutive proteolytic cleavages that lead to the production of Aβ, crucial in the etiology of AD. The basic biophysical and biochemical characteristics of the APP and Aβ generated in transgenic mice are significantly different from those of humans. The pathophysiology of AD is significantly more complex than the simple accumulation of Aβ [8]. Research utilizing animal models including transgenic rodents has produced inconsistent findings [9]. While the benefits of transgenic rodents are undeniable, further pertinent data on the physiopathology of AD can be gathered from other natural, non-transgenic models.

Could TWs be a new natural model of human disease, including NDDs? Can we name them Alzheimer’s or Parkinson’s? Are we faced with new NDDs affecting these marine mammals? It may be too early to discuss fully the neurobiological bases and underlying pathology of NDDs in TWs, but an attempt is made in this systematic review which examines all the literature published so far on this issue.

## Explored Potential Causes of Neurodegeneration in TWs

A total of 16 manuscripts have in some way addressed different aspects related to NDDs in cetaceans; the first was published in 2009 and the rest between 2017 and 2024 (Table 1). Five out of the 16 articles are commentary, letters to the editor, or short articles; four are from the same author, so are grouped and considered as one opinion article (Table 1). The manuscripts describe a total of 79 brains from specimens belonging to different shallow- and deep-diving TWs and different ages.

**Table 1.** List of literature that discussed the main conclusions and investigated the causes of NDDs in TWs.

Manuscript/type		Analyzed species, age, and sex	Main neuropathological finding(s)	Molecular studies		Explored cause(s)
1	[10] RA	1 BD; 1 RD; 1 SD Age and sex: unreported	Congo red-positive and Aβ-positive deposits in the brain, cerebellum, and medulla oblongata.	High amino-acid homology between APP, BACE, presenilin-1, and presenilin-2 in various dolphin species and the human counterpart; Aβ1-42 peptide 100% identical to the human peptide.		Aging
2	^[11]^ RA	8 animals*: BD; RD; SD Age and sex: reported only the age of one SD (adult)	Amyloid plaques were detected in three SD specimens, while NFTs were detected in four specimens (3 SD and 1 BD). Two different forms of amyloid deposit were described: diffuse deposits in the parietal cortex and formations in the cerebellum that resembled more compact senile plaques (ø $\sim$ 50 µm). Furthermore, 1 adult SD displayed distinct neurofibrillary threads in the frontal cortex.	The coding region of the APP gene containing the 40–43 amino-acid region of the Aβ peptide was identified by RNA and demonstrated to be the same in all three species and humans.		Extended postfertility life span and failure of insulin signaling /peripheral insulin resistance
3	^[12–15]^ C/LE	N/A	N/A	N/A		Cellular Prion Protein (PrPC) /Bioaccumulation and Biomagnification
4	^[16]^ CR	1 BD: Old ($\sim$ 40 years), male	Congophilic and Aβ-positive plaques; neuronal deposition of Αβ, cytoplasmic NFT immunostaining but no clear NFTs, and some neuronal immunoreactivity for ApoE in the parietal, frontal, and temporal lobes. Gliosis, microgliosis with degenerated neurons, chromatolysis, and some mild neuronophagia in the isocortex, cerebellum, and medulla.	–		Aging
5	^[17]^ RA	7 BD: 6 adults and 1 subadult; 4 females and 3 males 7 CD: 3 adults and 4 subadults; 3 females and 4 males	Aβ+ plaques and intracellular localization of Aβ in the cerebral cortex and brainstem of all stranded dolphins. Dystrophic neurites and neuropil threads in the auditory and visual areas of the cerebral cortex.	–		Bioaccumulation and Biomagnification/ Harmful Algal Blooms
6	^[18]^ RA	1 Fin whale (*Balaenoptera physalus*) (CBW): subadult, male 2 BBW: 1 adult, 1 subadult, females 1 S-FPW: adult, male 1 RD: adult, female 3 ASD: 1 old, 2 adults, males 1 BD: newborn, female	Six of nine showed Aβ immunopositivity in cerebral cortical neurons and/or NFT immunopositivity in cerebellar Purkinje neurons with granulovacuolar degeneration. Aβ plaques were also observed in one elderly animal.	–		Aging and Hypoxia
7	^[19]^ RA	MHW Neurons chemically reprogrammed from fibroblasts	N/A	Alteration of a number of genes involved in NDDs: ATP binding cassette subfamily A member 7 (ABCA7), ubiquitin-2 (UBQLN2), and Huntingtin (HTT), which are causative genes for AD, ALS, and HD.		Toxic/Epigenetic due to the exposition to a metabolite of PCBs
8	^[20]^ RA	7 CD; same animals as ^[17]^	Aβ+ plaques are associated with neuritic plaques, NFTs in the parietal and orbital lobes, and cerebellum. Widespread TDP-43 neuronal intracytoplasmic inclusions throughout all cortical layers. Widespread hypoxic-ischemic changes in neurons of the cerebral cortex and cerebellum.	Measurement of the gene expression of APP, PSEN1, PSEN2, MAPT, GRN, TARDBP, and C9orf72, using custom dolphin AD PCR assays.		Bioaccumulation and Biomagnification/ Harmful Algal Blooms
9	^[21]^ RA	1 ASD: adult, male 1 BBW: adult, female	First ultrastructural description of NM, resembling human NM.	–		Aging/Bioaccumulation and Biomagnification
10	^[22]^ RA	20 BD: 6 females and 14 males; 5 young adults ($\le$ 30 years old) 11 old adults ($\ge$ 30 years old) 4 calves	Aβ expression was investigated in the ventral cochlear nuclei (VCN) and inferior colliculi to detect acoustic trauma. One amyloid plaque in the white matter between the VCN and the superior olivary complex of one aged BD.	Western Blot established the specificity of the anti-Aβ antibody in the BD, SD, CBW, and sperm whale (*Physeter macrocephalus*)		Aging in the case of the Aβ plaque near the VCN
11	^[23]^ RA	2 BD: 1 male <1 year old, 1 male >30 years old) 5 HP: old; 4 females, 1 male 7 L-FPW: 5 old, 1 young adult, 1 subadult; 4 females, 3 males 2 RD: adult and old, females 6 W-BD: 3 old, 2 adult, 1 subadult; 3 females, 3 males	5 of the 18 adult/aged animals had one or more of the following changes: -Aβ-positive compact and diffuse amyloid plaques, vascular Aβ accumulations suggestive of CAA. - Phosphorylated Tau-positive pre-NFTs in the limbic and anterior paralimbic lobes; pTau-positive granules within neuronal cell bodies, axons, and dendrites, as well as accumulations consistent with pre-NFT.		–	Aging
12	^[24]^ C	N/A	N/A	N/A		Aging
13	^[25]^ RA	1 HP: subadult, female	The brainstem, midbrain, and cortex were all found to contain diffuse-type phospho-TDP-43 cytoplasmic inclusions. AD-type changes, including intracellular tangles, ghost tangles, dense-core bodies, granulovacuolar degeneration bodies (TDP-43+, Aβ+, and P62/SQSTM1+), and Hirano bodies were found in the hippocampus. Aβ+ plaques and intraneuronal staining in all brain regions.	In every brain region investigated from the HP, expression levels of all dementia-related genes (APP, PSEN1, PSEN2, GRN, MAPT; TARBDP, C9orf7) were found.		Bioaccumulation and Biomagnification/ Harmful Algal Blooms (BMAA toxin)

*BD* bottlenose dolphin/*Tursiops truncates*, *CD* common dolphin/*Delphinus delphis*, *HP* harbor porpoise/*Phocoena phocoena*, *RD* Risso’s dolphin/*Grampus griseus*, *SD* striped dolphin/*Stenella coeruleoalba*, *ASD* Atlantic spotted dolphin/*Stenella frontalis*, *BBW* Blainville’s beaked whale/*Mesoplodon densirostris*, *CBW* Cuvier’s beaked whale/*Ziphius cavirostris*, *L-FPW* long-finned pilot whale/*Globicephala melas*, *MHD* melon-headed whale/*Peponocephala electra*, *S-FPW* short-finned pilot whale/*Globicephala macrorhynchus*, *W-BD* white-beaked dolphin/*Lagenorhynchus albirostris*, *ALS* amyotrophic lateral sclerosis, *CCA* cerebral amyloid angiopathy, *HD* Huntington’s disease, *NM* neuromelanin, *N/A* not applicable, *C/LE* commentary/letter to editor, *CR* case report, *RA* research article, *not specified how many animals for each species

## Aging

Neurodegeneration has occasionally been reported in free-ranging cetaceans but it is most likely just underdiagnosed. The exhaustive study by Vacher *et al.* [23] on 22 brains from five different species of stranded TWs, reported that all the aged animals (15/22 specimens) had some kind of intraneuronal cytoplasmic and/or nuclear immunolabelling of Aβ in their supralimbic, paralimbic, and/or limbic lobes. Moreover, 5 animals, three aged long-finned pilot whales (*Globicephala melas*; family *Delphinidae*; deep-diver), one adult white-beaked dolphin (*Lagenorhynchus albirostris*; family *Delphinidae*; shallow-diver), and one aged bottlenose dolphin (*Tursiops truncatus*, Family *Delphinidae*; shallow-diver), had developed signs of AD-like pathology with amyloid plaques and/or intracellular granular phosphorylated-tau labelling, neuritic plaques, and in one long-finned pilot whale, inclusions resembling NFTs and neuropil threads. Conversely, AD-like disease was not found in any of the five harbor porpoises (*Phocoena phocoena*, Family *Phocoenidae*), a species that seldom survives longer than 13 years in the wild (source AnAge Database of Animal Aging and Longevity) [23]. Neurodegeneration might impact the behavior and cognitive abilities of elderly individuals due to Aβ deposits or other AD-like neuropathological characteristics; some authors suggest that this may have led to the stranding of these individuals followed by the youngest and healthiest ones [10]. As for other marine mammals, SPs and CAA have also been reported in an aged (~30 years) California sea lion (*Zalophus californianus*, *Pinnipedia*, Family *Otariidae*) [26]. Another study has shown that all the aged pinniped species examined (seal, sea lion, and walrus; families *Phocidae*, *Otariidae,* and *Odobenidae*, respectively) exhibit different degrees of AD-like pathology [6]. In general, the studies done so far in long-lived elderly -wild and captive- marine mammals have revealed signs of neurodegeneration in many species [16, 18, 23].

## The “Grandmother Hypothesis”

Gunn and co-authors direct attention to an important aspect, differentiating between longevity and aging [11]. Longevity is a complex phenotype that is impacted by environmental and genetic factors and is thereby the result of genes that have evolved because they allow an organism to reproduce [27]. Certain TWs have comparatively extended post-reproductive lifespans, which are essentially unique to humans among terrestrial mammals. According to the "grandmother hypothesis", having close-knit hunter-gatherer communities prepared for child care and other social tasks is advantageous for evolution [28]. On the other hand, extended post-reproductive lifespans result in a reduced efficiency of insulin and insulin-like growth factor signaling, a higher risk factor for AD and (pre)diabetes [11].

## Harmful Algal Blooms

A wide variety of species and classes of microalgae, including cyanobacteria, diatoms, and dinoflagellates, inhabit ecosystems from lakes to oceans, and are involved in harmful algal blooms (HABs), a varied phenomenon caused by the quick and exponential growth and accumulation of populations of these microalgae [29]. Aerosolized HAB toxins, like brevetoxins, okadaic acid, domoic acid, tetrodotoxin, saxitoxin, and ciguatoxin, have the potential to travel inland and cause harm to human health upon inhalation or exposure [30]. NDDs like amyotrophic lateral sclerosis (ALS) and AD have been linked to a newly identified toxin called β-N-methylamino-L-alanine (BMAA), which is a non-protein amino-acid produced by cyanobacteria [31]. Guamanian ALS/parkinsonism dementia complex, which is characterized by motor neuron degeneration and dementia, has been linked to BMAA consumption among the Chamorro community of Guam after eating their traditional food [32, 33]. The synergistic neurotoxicity of BMAA and methylmercury, which has been proposed to exacerbate dementia, must also be taken into account. In fact, 13 out of 14 dolphins had high levels of BMAA, 1.4 times greater than the brains of individuals with AD and ALS. In addition, all dolphins had Aβ+ plaques and intracellular Aβ localization in the cerebral cortex. BMAA has been identified as the cause of dystrophic neurites and Aβ plaques that accumulate in marine trophic chains [17]. AD-type changes linked to BMAA exposure have also been reported in a subadult pregnant harbor porpoise, including intracellular and ghost tangles, granulovacuolar degeneration bodies (TDP-43+, Aβ+, and P62/SQSTM1+), and Hirano bodies in the hippocampus, as well as Aβ+ plaques and intraneuronal staining in all brain regions [25]. Proteins linked to neurodegeneration have also been identified through proteomic analysis of cerebral fluid in California sea lions suffering from domoic acid toxicosis [34]. The underlying cause of the mass deaths of 12 rough-toothed dolphins (*Steno bredanensis*, Family *Delphinidae*) in the Canary Islands has also been attributed to brevetoxicosis; nevertheless, pathologic and neuropathologic analyses were hampered by the carcasses' mild to advanced decomposition [35]. Brevetoxins (polyether breve toxins; PbTxs) are lipid-soluble neurotoxins produced by dinoflagellates that affect fish, birds, mammals, and humans [36]. PbTxs cause acute neuronal injury and death [37] and neuronal degeneration [38].

## Hypoxia

Since Aβ and APP play a number of significant physiological roles in the regulation of synaptic activity and neuronal survival, they are not just pathogenic agents. Time-resolved research that links nuclear amyloid with neurodegeneration on the single-cell level also points to a protective role for nuclear amyloid, despite the basic hypothesis that it causes brain cell death [39]. Only Aβ-42 is involved in gene transcription, despite the fact that neuronal Aβ inclusions possess prion-like characteristics and can pass across the nuclear envelope *via* the nuclear pore complex to reach the nucleus [40, 41]. The nuclear localization of Aβ may contribute to modifications in DNA topology by influencing the helicity and superhelicity of supercoiled DNA [42]. One of the manuscripts showed intranuclear neuronal Aβ production in two animals, a subadult Cuvier’s Beaked Whale (*Ziphius cavirostris*, Family *Ziphiidae*, deep-diver) stranded in close temporal and geographic association with an international naval exercise. In this case, intranuclear neuronal Aβ production has been proposed as a neuroprotective reaction against hypoxia. The other was an elderly Atlantic spotted dolphin (*Stenella frontalis*; family *Delphinidae;* shallow-diver) which also showed SPs [18]. Another study examined the ventral cochlear nuclei of 16 bottlenose dolphins (shallow diver); 14 of them exhibited an intense Aβ intranuclear immunoreactivity while one, which asphyxiated in a net, did not. In addition, intranuclear neuronal Aβ was substantially more intense and extensive in calves than in old animals. Similarly, the authors propose that intranuclear Aβ is a neuroprotective mechanism against neuronal death [22]. Intense Aβ intranuclear labeling was seen in a small to medium number of neurons of the 15 aged and 3 adult TWs (shallow and deep divers) examined by Vacher *et al.* (2022); intraneuronal labelling was present in 3 out of the 4 younger animals, used as species control references [23]. The brains of deep-diving TWs, mainly beaked whales but also sperm- and pilot-whales, may be susceptible to neurodegenerative changes as a result of sustained hypoxia [18]. With a dive at 3000 meters lasting 2h, a Cuvier's beaked whale broke the record for the deepest dive ever recorded by an air-breathing endotherm [43]. The duration and degree of hypoxia in various cells determine whether the hypoxia has a positive or negative impact on the cells. Because hypoxia affects the processing of APP, it has been identified as a risk factor that may accelerate the onset of AD. Also, recurrent hypoxia promotes the production of Aβ and neuritic plaques [44]. Despite their adaptations, beaked whales are unfortunately vulnerable to repeated episodes of protracted cerebral hypoxia since their dives and feeding activity are frequently interrupted by increasing anthropogenic noise [45], as in international naval exercises.

Hypoxia has then been proposed as one of the most important risk factors that may accelerate the pathogenesis of NDDs by altering APP processing. Furthermore, recurrent hypoxia triggers macroautophagy and enhances the production of amyloid and neuritic plaques [44].

While the brain has been shown to undergo protective adaptations in response to mild, moderate, and/or intermittent hypoxia [46], severe and/or persistent hypoxia can cause inflammation, reactive oxygen species (ROS) production, and impact cellular metabolism [47]. Moreover, the aging brain exhibits several modifications, including decreased cerebral blood flow, alterations in white matter, iron overload, and neuroinflammation [48]. One of the common vascular components among AD risk factors is cerebral hypoxia, which is caused by a reduction in cerebral blood flow that results in hypoperfusion [49]. In humans, vascular pathology is evident in one-third of AD patients, indicating a significant vascular component that may contribute to brain injury. With aging, a mixed etiology of AD and vascular disease is predicted to become increasingly prevalent [50].

## Bioaccumulation and Biomagnification: Environmental Medicine, Epigenetics, and Planetary Health as Key Points to Understand NDDs

The bioaccumulation and biomagnification of organic and metallic contaminants pose serious threats to TWs, as their blubber (a thick layer of subcutaneous adipose tissue) serves as a depot for toxic compounds. *Environmental Medicine* is a specialized field of clinical medicine and is dedicated to the worldwide, comprehensive, and translational study of the effects of the environment on humans [51]. Secular effects (diet, place of residence, education) are critical factors for both health and disease. Furthermore, the health of every species on this planet is impacted by pollution. Whatever their effects, animals are susceptible to the same symptoms and illnesses as people. *Planetary Health* refers to the state of the natural systems that support our species, such as the biosphere's diversity and health [52]. Climate change, ocean acidification, and warming, chemical pollution, and greenhouse gas emissions are only some of the disastrous consequences of ruthless human activity. Native fauna may suffer harm from various sources including water toxicity, decreased oxygen content in the lower layers of water bodies, and challenges in adjusting to novel compounds. Forest area loss has increased the risk of zoonotic illness by bringing people and wildlife into closer contact [52]. Anthropogenic contaminants are substances produced by industrial and agricultural operations that are poorly digested, can linger in the aquatic environment, and can be harmful [53]. Hazardous trace elements, formerly known as heavy metals, and persistent organic pollutants (POPs), which are industrial products, are two types of anthropogenic contaminants. Due to human activity, POPs and trace elements are more common in nature. POPs were formerly used as industrial chemicals, solvents, insecticides, and medications. They are resistant to natural processes and chemical deterioration [54]. Organic toxicants [(polychlorinated biphenyls (PCBs), organochlorine pesticides, polycyclic aromatic hydrocarbons, atrazine, diethyl phthalate, nonylphenol monoethoxylate, and triclosan)], non-essential elements (arsenic, cadmium, lead, mercury, thallium), essential elements (cobalt, copper, manganese, iron, selenium, zinc), and/or one toxicant mixture class, Aroclor1268, have been detected in TWs [53, 55–58]. Decades of local industrial activity have resulted in the discharge of a highly chlorinated PCB combination, known as Aroclor 1268, into the aquatic environment [59].

Among the worst and most widespread contaminants in the environment are plastics [51]. Plasticizers [phthalates, bisphenol-A (BPA), nonylphenol ethoxylates, PCBs, and organochlorines] are a class of xenobiotics acting as endocrine-disrupting compounds and can have a negative impact on a variety of wildlife species' organ systems [60].

A large cohort retrospective study of 465 cetaceans from 15 distinct species, stranded in the Canary Islands between 2000 and 2015, revealed that at least one ingested foreign body was found in the gastrointestinal tract of almost 8% of the animals analyzed. Almost 60% of them were deep diver species and nearly 81% of the items found were plastics [61]. An up-to-date comprehensive study has yielded very interesting data and conclusions about the plastic uptake by two cetacean families, the beaked whales (family *Ziphiidae*) and the family *Delphinidae*. Due to their non-selective suction-feeding method, beaked whales showed a higher presence of microplastics in their digestive system than dolphins. Moreover, microplastics are immediately inhaled by cetaceans at the air-water interface, and they are subsequently ingested through trophic transfer from prey [62]. Even more worrying are the problems arising from microplastics. A study conducted on the intestines of 38 small TWs stranded on the coast of Portugal revealed that >90% of the individuals analyzed had microplastics in their intestines [63].

In humans, different hazardous contaminants, such as plasticizers (phthalate esters, Bisphenol A), pesticide residues, flame retardants, heavy metals, and even air pollution (including secondhand smoke) affect neurodevelopment and neurodegeneration through a variety of pathophysiological mechanisms, such as mitochondrial damage, oxidative stress, cell death, neurotransmitter dysregulation, endocrine disruption, and epigenetic modification [64, 65].

Recent research used both wild-type and AD-predisposed mice to investigate the pathogenic effects of air pollution particles on AD and their molecular relationships. In AD-predisposed mice, there was an increase in the detection of Aβ plaque and a loss of hippocampal and somatosensory cortical neurons (wild-type mice lack the genetic predisposition to create the plaque) [66].

On the other hand, the study of heritable changes in gene activity brought about by causes other than DNA sequence alterations is known as *epigenetics*. Studying changes in DNA methylation, DNA-protein interactions, chromatin accessibility, histone modifications, and other processes is one aspect of epigenetic analysis. Exposure to plasticizers may modify histones, which can lead to epigenetic changes. Autophagy, protein aggregation, and epigenetic modifications are significant cellular and molecular indicators of NDDs brought on by long-term exposure to neurotoxic chemicals [67]. Epigenetic alterations have been demonstrated in neurons, chemically reprogrammed from fibroblasts from mass-stranded melon-headed whales (*Peponocephala electra*, family *Delphinidae*), due to exposure to a metabolite of PCBs. In addition, exposure to PCBs has been linked to neurodegeneration through disrupted apoptotic processes [19].

Ultimately making everything worse, POPs, pesticides, heavy metals and other contaminants accumulate in milk [68]. This is particularly important for TWs having very long breastfeeding periods, as a transfer from female to calf has been demonstrated in the bottlenose dolphin [69]. Bioaccumulation and biomagnification in TWs are particularly critical as they are at the top of the marine food web and must be taken into account when studying their health. The marine food web projects carnivorous marine mammals, such as TWs, into the pole position of the marine animals at the highest risk from oceanic contamination. Since the TWs are regarded as "sentinels" for the marine environment, they are particularly useful as disease models to better understand the impact of contaminants on our health.

## Neuronal Selective Vulnerability

Well-established spontaneous animal models of NDDs cannot be configured with just two parameters (Aβ and NFT). Neuromelanin (NM) has been described in neurons of different animal species like horses, giraffes, cattle, sheep, goats, dogs, rats, and even in frogs and tadpoles (*Rana esculenta*) [70–74]. The ultrastructure of NM in most of these animals is similar to lipofuscin, with a typical lamellar pattern. On the other side, NM in humans and primates exhibits particular traits, not found in other animals. NM has been reported for the first time in different species of the family *Delphinidae* [75] and then recently characterized in two cetacean species [21]. In fact, an Atlantic spotted dolphin and a Blainville's beaked whale (*Mesoplodon densirostris*; family *Ziphiidae*) were found to have melanin granules associated with lipid droplets and membranes, morphologically similar to human NM. The idea that PD is unique to humans was underscored by the discovery of a real NM in these two species; nevertheless, other species like TWs should be investigated. NM-containing neurons uniformly deteriorate in Parkinson’s disease (PD) across a variety of brain areas, such as the substantia nigra pars compacta and the noradrenergic neurons of the locus caeruleus [76]. When NM accumulates above a threshold, its progressive intracellular warehousing appears to endanger neuronal function, triggering activation of microglial (rod) cells, followed by eventual neuronophagia, and/or Lewy body formation [77]. Our understanding of selectively vulnerable regions of the brain is a key point to disentangle NDDs. Neuronal vulnerability is especially important under hypoxic or toxic conditions. Both the Purkinje cells of the cerebellum and neurons of the hippocampus are susceptible to hypoxia [78]. Granulovacuolar degeneration (GVD) has been observed as NFT-positive granules, in the Purkinje neurons of the anterior and posterior cerebellum of two Blainville's beaked whales and one Atlantic spotted dolphin. GVD is interpreted as either a cellular defense mechanism or an indicator of impaired cellular functioning [79]. GVD is one of the histopathological hallmarks of AD [80] and its granules are often immunoreactive for hyperphosphorylated tau and phosphorylated TDP-43 [81]. In 7 short-beaked common dolphins (*Delphinus delphis*; family *Delphinidae*) examined for exposure to BMAA and Methylmercury, widespread neuronal inclusion bodies, immunopositive for TAR DNA-binding protein 43 (TDP-43) were recognized in the cerebral cortex, while cerebellar Purkinje neurons showed chromatolysis and vacuolization [20]. In a subadult pregnant harbor porpoise, diffuse-type phosphor-TDP-43 cytoplasmic inclusions have been identified in the brainstem, midbrain, and cortex. In addition, phosphor-TDP-43 GVD has been found in the hippocampus [25]. Pathological TDP-43, which includes hyperphosphorylated, ubiquitinated, and/or cleaved forms, is involved in the disease mechanisms of both ALS and ubiquitin-positive, tau- and alpha-synuclein-negative frontotemporal dementia [82]. Hippocampal TDP-43 pathology has been proposed as a contributor to necrosome-positive GVD in ALS and frontotemporal dementia [83]. Future research is needed to explore the mechanisms driving TW-selective neural vulnerability and GVD.

## Other Considerations

Numerous patients with a clinical diagnosis of AD have undergone neuropathological investigations that revealed the hallmark lesions of PD, namely the neuronal degeneration of the substantia nigra pars compacta with Lewy bodies in many of the remaining neurons [84]. A disease known as mixed dementia occurs when alterations in the brain from many dementia causes occur at the same time. The most frequent forms include AD and blood vessel problems linked to vascular dementia, AD with Lewy bodies, and AD with vascular problems and Lewy bodies. Non-viral inclusions bodies are barely reported in the literature in marine mammals. Pale basophilic cytoplasmic inclusions encircled by a rim of NM granules have been found in a Blainville's beaked whale [21]. Pale bodies, the precursor to Lewy bodies, are found in neurons containing NM and are linked to Lewy pathology. The Lewy pathology is caused by an insoluble form of the protein called α-synuclein. Alpha-synuclein is redistributed to NM pigment in the early stages of PD and becomes lodged inside NM granules [85]. Alpha-synuclein- and ubiquitin-positive inclusions have been reported in the midbrain of a short-finned pilot whale (*Globicephala macrorhynchus*, family *Delphinidae*) [86].

Lastly, a crucial facet of the pathophysiology of AD and PD is the dysregulation of microglia [87, 88]. In the articles published so far, microglia have barely been reported in animals with AD-like lesions [23]. It is important to remember, though, that activated microglia are frequently seen in stranded cetaceans (based on personal observation), frequently as a result of underlying infectious diseases [89, 90].

From this perspective as well, TWs may be a very useful research resource for the study of microglial activation, as well as the hereditary forms of AD and PD and the impact of genetic susceptibility factors [91].

## Some Neuroprotective Factors Against NNDs in Cetaceans

### Sharks, Turtles, and Baleen Whales: What Do They Have in Common?

A highly startling study revealed the structural characteristics of a ~245 ± 38-year-old female Greenland shark's brain, which was collected in a bottom trawl deep off West Iceland [92]. It was remarkable that, despite this elasmobranch fish's old age, no macroscopic or microscopic evidence of neurodegeneration could be seen in its brain. The authors considered the specific physiological characteristics of this animal as protective factors, such as a low aerobic metabolism, little mitochondrial oxidative stress, and high concentrations of trimethylamine, which may be neuroprotective, as well as specific environmental factors, such as the fact that Greenland sharks live primarily in cold (4°C) waters deep in the Arctic ocean with remarkably slow movements. In addition, it has been hypothesized that Greenland sharks have comparatively low blood pressure in comparison to other sharks, which may lower the risk of hypertension-related brain harm like stroke or cognitive decline. Some turtles are shielded from brain damage when they emerge from underwater hibernation because they produce less mitochondrial ROS [93]. ROS are produced during the hypoxic period or after re-oxygenation. In fact, when oxygen returns to the brain after a stroke, ROS are released, which causes brain damage. The review by Lagunas-Rangel recollects important aspects of the longevity of baleen whales, reaching in some species a life expectancy of >100 up to a recorded 211 years [94]. The author suggests using whales as natural models for anti-aging and cancer resistance after revealing many traits, including their basal metabolism, the body temperature (low in some baleen whales like the bowhead whale), lower ROS production during hypercapnia, or the quality of their genome (including high expression of protective genes against aging and cancer). Whales maintain extremely high levels of neuroglobin mRNA in their brains, ensuring that oxygen is stored and can detoxify ROS. Higher neuroglobin levels are therefore advantageous because they would guard against ROS and enhance the oxygen supply throughout dives [95]. Neuroglobin supports the basic hypoxic tolerance of the diving brain by scavenging reactive oxygen and nitrogen groups and averting cellular harm [95, 96]. It is crucial to emphasize that despite all of their adaptive phenomena and advantageous environmental traits, NDDs are still present in cetaceans. It is also accurate to note that baleen whales have not yet been found to exhibit any indication of NDDs. In fact, all the research until now has focused on TWs, the highest top predators, while the diet of baleen whales relies on zooplankton and small fish or copepods.

**Diet:** polyunsaturated fatty acids (**PUFAs) and other neuroprotective substances**

Healthy nutrition is crucial for the purpose of promoting good cognitive aging and reducing the risk of dementia [97]. Marine compounds do not merely comprise toxins but they include substances like some peptides, pigments, lipids, glycosaminoglycans, or polysaccharides present in marine flora and fauna with strong neuroprotective actions [98]. In the field of environmental medicine, one can question why animals living in environments naturally enriched with PUFAs would experience NDDs. PUFAs are essential for maintaining healthy cell membranes, operate as signaling molecules, control inflammatory reactions, and provide an important source of energy [99]. The ecosystem in which cetaceans, especially TWs, dwell is naturally abundant in Omega-3 (ω-3) PUFA. Fish and squid contain ω-3 PUFA, which is essential to human metabolism. As precursors to arachidonic acid and docosahexaenoic acid, linoleic acid, and α-linolenic acid are absorbed through the diet [100]. ω-3 PUFA is particularly abundant in the brain. PUFA reduces Aβ-amyloid toxicity, has anti-aggregation properties, and inhibits Aβ40 and Aβ42 fibrillogenesis [99].

### Other Considerations

On the other side, it would be very interesting to know if the pathology we find in these animals has an impact on cognition and behavior. In human medicine, individual variations in task performance that may enable some persons to be more resilient than others are referred to as cognitive reserve. The prevention of cognitive deterioration is aided by cognitive reserve [101]. Lifestyle experiences, such as accomplishments in school and the workplace, hobbies, or language skills (bilingualism/multilingualism) contribute to the development of cognitive reserve [102]. Cognitive reserve counteracts the effects of aging, age-related diseases, and NDDs. It is improbable that these kinds of research could be carried out on wild TWs, due to their protected status and the difficulty of conducting *in vivo* studies, the majority of which are performed on deceased subjects. However, it is possible to determine the relationship between NDDs and behavior by attempting to apply this research to captive dolphins; many elements have been suggested as having an impact on welfare outcomes including habitat features, environmental enrichment initiatives, and training initiatives [103].

## Conclusions

**Fig. 2:**
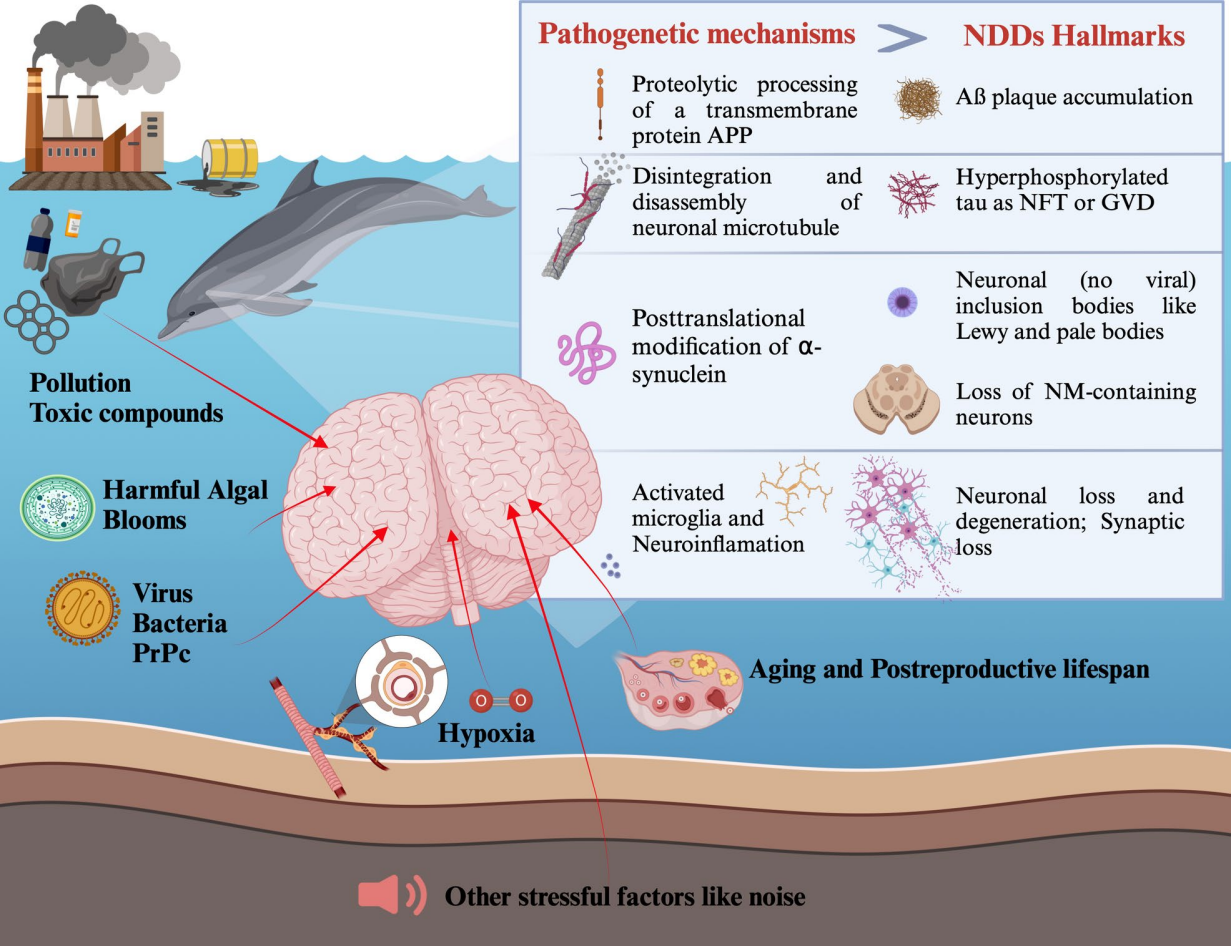
Infographic of possible causes of neurodegeneration in TWs and consequent hallmarks. Created with Biorender.com, last accessed on 22 May 2024.

Are TWs therefore spontaneous animal models of NDDs? According to this review, they are, hence the hypothesis is summarized here. Cetaceans are “sentinels” for the sea environment and thus represent valuable disease models for their human (and animal) counterparts. Both humans (terrestrial mammals) and marine mammals live in a highly contaminated context and are continually subjected to stressors of different natures (such as pollution, noise, and food). Numerous environmental factors that affect TWs can change their behavior and shorten their longevity. Surely, NDDs are the price both humans and TWs pay for the molecular underpinning that makes it possible for humans and TWs to live almost unmatched long lives and to be exposed to various but shared danger factors (Fig. 2). The neuropathological features of NDDs represent a final common pathway of disease with multiple influences including in this context aging but also other risk factors. TWs might also show atypical hallmarks of NDDs. Although data are limited, there appear to be further similarities between humans and TWs, which could help pinpoint additional commonalities in the development of NDDs like sporadic AD. Aged TWs housed in captivity in zoos or aquariums could also help shed more light on this topic.

Finally, a standardized protocol for brain collection should be included in research on neuroanatomy and neuropathology, including neurodegeneration. This protocol should be supplemented by different neuromarkers that are useful for a comprehensive diagnosis of NDDs. The goal is to raise awareness of the NNDS among pathologists involved in marine mammal research and the biomedical community overall.
